# Study of the Characterization of Side Population Cells in Endometrial Cancer Cell Lines: Chemoresistance, Progestin Resistance, and Radioresistance

**DOI:** 10.3389/fmed.2020.00070

**Published:** 2020-03-18

**Authors:** Bing-jie Liu, Qi-ying Xu, Wei-dong Yu, Na Li, Tian Yao, Li-jun Zhao, Jian-liu Wang, Li-hui Wei, Xiao-ping Li

**Affiliations:** ^1^Department of Gynecology, Peking University People's Hospital, Beijing, China; ^2^Qinghai University Affiliated Hospital, Xining, China; ^3^Central Lab, Peking University People's Hospital, Beijing, China

**Keywords:** endometrial cancer, side population cells, chemoresistance, radioresistance, breast cancer resistance protein (BCRP)

## Abstract

**Introduction:** Radiotherapy, combined regimens as platinum-paclitaxel chemotherapy and/or endocrine therapy is an important adjuvant treatment after surgery for endometrial cancer (EC). While, the resistance to them remain unclear. In our study, to separate the characteristics of side population (SP) cells from EC cell lines, study the mechanism of Taxol-resistance, progestin resistance and radioresistanc, and provide the basic for EC.

**Methods:** SP cells from EC cell lines HEC-1A, Ishikawa and RL95-2 were separated by Hoechst 33342 staining and flow cytometry analysis. The expression of breast cancer resistance protein (BCRP) in SP cells and non-SP cells from HEC-1A was examined by immunocytochemistry, and the radiation-resistant and Taxol-resistant characteristics of SP cells and non-SP cells were compared by MTS. Ishikawa, Ishikawa-SP, and Ishikawa-non-SP cells incubated with MPA were selected for cell apoptosis assays by using flow cytometry. The expression of caspase-3 was examined by immunocytochemistry, and autophagy was detected by MDC staining.

**Results:** Small proportions of SP cells, namely, 1.44 ± 0.93%, 2.86 ± 3.09%, and 2.87 ± 1.29%, were detected in HEC-1A, Ishikawa and RL95-2, respectively. There was a stronger clone formation efficiency for the SP cells than for non-SP cells in HEC-1A [(6.02 ± 1.17) vs. (0.53±0.20)%, *P* = 0.001], and there was a significant difference in the rate of tumourigenicity between the SP cells and non-SP cells in HEC-1A (87.5 vs. 12.5%). There were higher levels of BCRP expression (*P* = 0.001) and resistance to Taxol and radiation (*P* < 0.05) in the SP cells than in non-SP cells. After MPA treatment, the apoptosis rates were significantly different among the Ishikawa, Ishikawa-SP and Ishikawa-non-SP groups [(4.64 ± 0.18)%, (4.01 ± 0.43)%, and (9.3 ± 0.67)%; (*P* = 0.05)], and the expression of Caspase-3 in the Ishikawa group was higher than that in Ishikawa-SP group. The autophagic activity of the Ishikawa-SP cells was the strongest, while the autophagic activity of Ishikawa-non-SP was the weakest.

**Conclusions:** There is a significant enrichment in SP cells among different EC cell lines, and these SP cells be more resistant to Taxol, MPA and radiation therapy. The overexpression of BCRP among SP cells may be the cause of resistance to Taxol, progestin and radiotherapy, which may be related to apoptosis and autophagic activity.

## Introduction

Endometrial cancer is one of the most common malignant tumors in women, and it is the most common malignant tumor of the female reproductive tract in European and American developed countries (1). In addition, the incidence rate is increasing, and the onset age is younger in China. Although radiotherapy, chemotherapy and/or endocrine therapy after surgery have achieved good relief and survival, these approaches still have risks for tumor recurrence, metastasis, chemoresistance, progestin resistance, and/or radioresistance. So, understanding the disease pathogenesis, overcoming its chemoresistance, progestin resistance, and/or radioresistance have important significance.

According National Comprehensive Cancer Network, Platinum-based combined regimens as platinum-paclitaxel (TC) is usually used chemotherapy regimen for advanced and recurrent endometrial cancer. Paclitaxel (Taxol) is a first-line chemotherapeutic drug for gynecological malignancies and exerts anti-tumor effects through various mechanisms, such as blocking the mitosis of tumor cells and inducing apoptosis. There were many studies of Cisplatin-resistant in endometrial cancer. While, there were few studies have focused on the mechanism of paclitaxel-resistance in endometrial cancer. Therefore, whether there is paclitaxel- resistance in endometrial cancer cells and its mechanism is our focused in the study.

The cancer stem cell hypothesis has led to a new theory in recent years. It is suggested that there is a small number of stem cells in the tumor, which may be play a decisive role in the formation, growth, invasion and metastasis of the tumor (2–4). Further studies have shown that these cells may also be resistant to radiation therapy and chemotherapy (5). Therefore, tumor stem cells will become hot topics of research and therapeutic targets in the future. There are few reports on endometrial cancer stem cells, especially the study between endometrial cancer stem cells and chemoresistance, progestin resistance or radioresistance.

Currently, there are two main methods to separate tumor stem cells: the first selects and separates cells based on the surface-specific markers of tumor stem cells (6–8), and the second selects and separates a small group of a Hoechst33342 tropochrome “side population” (SP) cells from the tumor cell line by flow cytometry, with the characteristic of higher ABC transport protein expression in tumor stem cells (9, 10). Because there is a lack of surface-specific markers for tumor stem cells, the method to separate SP cells has become the main method which used to study tumor stem cells. Therefore, this study was designed to use this method to separate SP cells from endometrial cancer cell lines and preliminarily study the chemoresistance, radioresistance, and progestin resistance characteristics of these cells, as well as provide a basis for clinical treatment and provide a basis strategy for the study of drug resistance.

## Materials and Methods

### To Separate and Identify SP Cells and Investigate Their Characteristics

Human endometrial cancer cell lines included Ishikawa [ERα(+), ERβ(+), PR(+)], HEC-1A [ERα(+), ERβ(–), PR(+)], and RL95-2 [ERα(+), ERβ(–), PR(+)] all of which were preserved in the Obstetrics and Gynecology Laboratory, Peking University People's Hospital. SP and non-SP subsets were sorted by flow cytometry (9). After the separation, the SP and non-SP cells of the HEC-1A cell line were used to investigate growth characteristics and tumourigenicity *in vitro* and *in vivo*.

### BCRP Protein Expression in SP and Non-SP Cells of the HEC-1A Cell Line as Detected by Immunocytochemistry

The SP and non-SP cells of the HEC-1A cell lines were separately seeded onto glass slides, fixed with paraformaldehyde for 30 min, washed with PBS, and incubated with BCRP monoclonal antibody at a 1:100 dilution. Simultaneously, an anti-dilution solution was used as the negative control; cells were incubated at 4°C overnight, washed with PBS, incubated in 45–50 μl of universal IgG-HRP antibody, placed at room temperature for 30 min, washed with PBS, and subjected to conventional DAB staining, dehydration, mounting, and sealing. Finally, the cells were observed under a microscope. For each sample, 10 high-power lens fields were randomly selected, and 10 cells were counted in each field for grading according to the coloring depth of the cell as follows: no coloring was 0 points, light yellow was 1 point, brown was 2 points, and dark brown was 3 points. The number of colored cells was counted, and the scores were obtained by integral calculation. BCRP was located in the cell membrane, and the staining was the brownish yellow.

### Sensitivity of the SP and Non-SP Cells of the HEC-1A Cell Line to Taxol as Detected With the MTS Method

The SP and non-SP cells of the HEC-1-A cell line were separated by flow cytometry, and HEC-1A cells without any treatment were used as the control group, and the cells density was adjusted to 3 × 10^5^ cell/mL. Cells were inoculated in 96-well plates. Each cell type was divided into 7 groups, and each group had five wells. At the same time, the zero well (i.e., the blank group) was included (only with medium) with a volume of 100 μl/well. The cells were placed in a humidified incubator at 37°C with 5% CO_2_ for culturing, and when the cells were attached, the medium was changed to 100 μl culture medium containing different Taxol (TAX) concentrations (drug concentrations were 1, 4, and 6 μg/mL). The medium of the blank group and control group was changed to fresh culture medium without TAX, and after culturing for 24 h, the number of live cells was determined with MTS method.

### Sensitivity of the SP and Non-SP Cells of the HEC-1A Cell Line to Radioactive X-Ray Irradiation as Detected With the MTS Method

The SP and non-SP cells of the HEC-1A cell line were separated by flow cytometry, and HEC-1A cells without any treatment were used as the control group. Each cell type was divided into five dosage groups, namely, 0 Gy (i.e., the group without exposure to X-rays), 1, 2, 4, and 6 Gy (the high-energy X-ray irradiation groups). Irradiation methods were as follows: the linear accelerator was used, the fixed distance from the source to the skin was 100 cm, the dosage rate was 4 Gy/min, and the range of irradiation was set to cover the culture dish. After irradiation, cells were seed to 96-well plates. Each well had 2 × 10^3^ cells, and each group had five wells. At the same time, the zero well (i.e., the blank group) and control group were included (only with medium). The MTS method was used to determine the growth curve of the cells, as described above.

### Sensitivity of the SP and Non-SP Cells of the Ishikawa Cell Line to MPA as Detected With the MTS Assay

SP and non-SP cells if the Ishikawa cell line were separated by flow cytometry, and Ishikawa cells without any treatment were used as the control group. The cell density was adjusted to 1 × 10^5^ cell/mL, and cells were inoculated into 96-well plates. Each cell type was divided into 5 groups, and each group had five wells. At the same time, the zero well (i.e., the blank group) was included (only with medium) at 100 μl/well. The cells were placed in a humidified incubator at 37°C with 5% CO_2_ for culturing, and when the cells had attached, the medium was changed to 100 μl of culture medium containing different MPA concentrations (drug concentrations were 0, 5, 10, 15, and 20 μmol/L), and the medium of the blank group and control group was changed to fresh culture medium without MPA. After culturing for 24, 48, and 72 h, the number of live cells was determined with the MTS method, and the inhibitor rate was calculated as follows: Inhibitor rate = (A_control_ – A_experiment_)/A_control_ × 100%.

### Apoptosis of SP Cells in Response to MPA as Detected by Flow Cytometry

SP and non-SP cells in Ishikawa cell lines were separated by flow cytometry, and Ishikawa cells without any treatment were used as the control group. After inoculation into in 6-well plates, the cells were placed in a humidified incubator at 37°C with 5% CO_2_ for culturing, and when the cells had attached, the medium was changed to 2 mL of culture medium containing 10 μM MPA, and to fresh culture medium without MPA for the control group. After culturing for 48 and 72 h, the cells were collected, washed twice with cold PBS and resuspended in 1X Binding buffer at a concentration of 1 × 10^6^ cells/mL. A total of 100 μl of the solution was transferred to a 5-mL culture tube. Five microliters of FITC Annexin V and 5 μl PI were added, and the cells were gently vortexed and incubated for 15 min at 25°C in the dark. A total of 400 μl of 1X binding buffer was added to each tube, and cells were analyzed by flow cytometry.

### Caspase-3 Protein Expression in the SP Cells of the Ishikawa Cell Line in Response to MPA as Detected by Immunocytochemistry

The SP and non-SP cells of the Ishikawa cell line (Ishikawa cell line as control) treated with MPA were separately seeded onto glass slides, fixed with paraformaldehyde for 30 min, and washed with PBS, and then caspase-3 monoclonal antibody was added at a 1:100 dilution. An anti-dilution solution was used as the negative control, and the cells were incubated at 4°C overnight and washed with PBS. Then, 45–50 μl of universal IgG-HRP antibody was added to the cells, and the cells were placed at room temperature for 30 min, washed with PBS, and subjected to conventional DAB staining, dehydration, mounting, and sealing. Finally, cells were observed under a microscope. For each sample, 10 high-power lens fields were randomly selected, and 10 cells were counted in each field for grading according to the coloring depth of the cells as follows: no coloring was 0 points, light yellow was 1 point, brown was 2 points, and dark brown was 3 points. The number of colored cells was counted, and the scores were obtained by integral calculation.

### Statistical Analysis

Each measurement result was expressed as the mean ± standard deviation ($\bar{\text{x}}$ ± s). SPSS 13.0 software was used for data analysis, and comparisons between two groups were analyzed with the *t* test. A *P* < 0.05 (*P* < 0.05) indicated a significant difference.

## Results

### To Separate and Identify SP Cells and Investigate Their Characteristics

#### Proportion of SP Cells in the Three Kinds of Cell Lines: Human Endometrial Cancer HEC-1A, Ishikawa and RL95-2

Hoechst 33342 staining was performed, and the proportions of SP cells in HEC-1A, Ishikawa and RL95-2 detected by the flow cytometry were 1.44 ± 0.93, 2.86 ± 3.09, and 2.87 ± 1.29%, respectively.

#### Morphological Observation of SP Cells in Ishikawa, HEC-1A, and RL95-2

**Figure 1:**
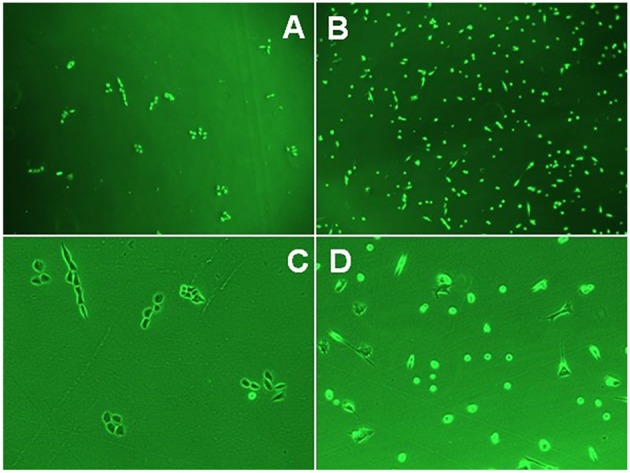
**(A)** HEC-lA-SP cells (10X). **(B)** HEC-lA-non-SP cells (10X). **(C)** HEC-lA-SP cells (40X). **(D)** HEC-lA-non-SP cells (40X).

The SP and non-SP cells separated from Ishikawa, HEC-1A, and RL95-2 were cultured for observation every 6 h under an inverted microscope. The volumes of SP cells were smaller than those of non-SP cells, and SP cells were much more easily attached than non-SP cells. Twenty-four hours after inoculation, most of the SP cells were attached, showing colony growth, while the number of attached non-SP cells was significantly lower than that of attached SP cells. Cell morphology images of the SP and non-SP of the HEC-1-A cell line are shown in Figure 1.

#### Determination of the Growth Curves of HEC-1A-SP and HEC-1A-non-SP Cells

**Figure 2:**
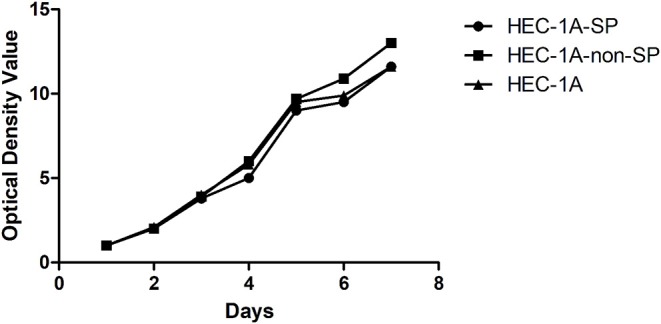
The Growth curve of HEC-1A-SP, HEC-1A-non-SP, HEC-1-A.

The HEC-1A-SP, HEC-1A-non-SP, and control HEC-1A cells were cultured for 7 days and grown to confluence. The doubling times of the HEC-1A-SP, HEC-1A-non-SP, and control HEC-1A cells were measured with the MTS method as follows: 47.17 ± 2.04, 44.62 ± 0.91, and 48.48 ± 1.07 h, respectively. The results are shown in Figure 2.

#### Results of the Monoclonal Formation Experiment of the SP and Non-SP of the HEC-1A Cell Line

The separated SP and non-SP cells from the HEC-1A cell line were cultured for 14 day. The two groups of cells were grown in 6-well plates and formed clones. The rate of clone formation of the SP cells was (6.02 ± 1.17)%, while that of the non-SP cells was (0.53 ± 0.20)%. There was statistical significance in the difference between the CFE of the two groups (*P* = 0.001). In the SP group, seven nude mice had tumors at 3–4 weeks after inoculation (87.5%, 7/8), while only one mouse in the non-SP group had a tumor at 6 weeks after inoculation (12.5%, 1/8). Additionally, the size of the tumor was significantly smaller than that of the SP group.

### Determination of BCRP Protein Expression in HEC-1A-SP and HEC-1A-non-SP Cells and the Cell Sensitivity to Taxol

#### Determination of BCRP Protein Expression in HEC-1A-SP and HEC-1A-non-SP Cells

**Figure 3:**
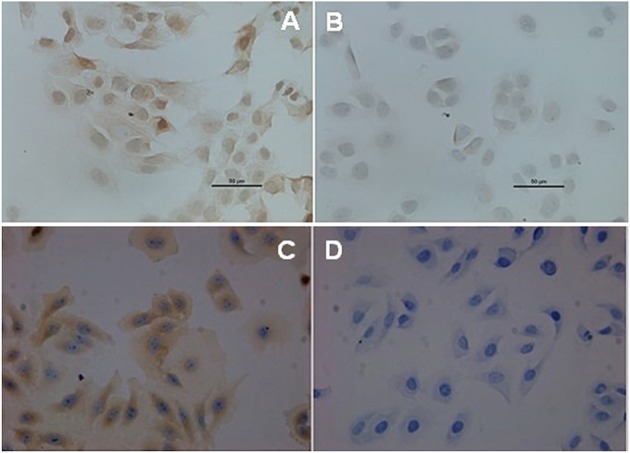
BCRP expression in SP and non-SP cells. **(A)** SP cells (40X). **(B)** Non-SP cells (40X). **(C)** MCF-7 positive control (40X). **(D)** MCF-7 negative control (40X).

The immunocytochemistry staining results showed that there was BCRP expression in the cell membrane and cytoplasm of HEC-1A-SP and HEC-1A-non-SP, but BCRP expression in SP cells was significantly stronger than that in non-SP cells (*P* = 0.001), as shown in Figure 3.

#### Determination of the Sensitivity of HEC-1A-SP and HEC-1A-non-SP Cells to Taxol

After different concentrations (1, 2, and 4 μg/mL) of Taxol were added, the survival rates of HEC-1A-SP, HEC-1A-non-SP, and HEC-1A were decreased and showed dose dependence. The MTS results suggested that the SP cells might have Taxol resistance compared with the control HEC-1A cells and non-SP cells. The results are shown in Table 1.

**Table 1:** Anti-TAX characteristics of endometrial cancer HEC-1A, HEC-1A-SP, and HEC-1A-non-SP cells.

**Item**	**TAX (μg/mL)**		
	**1**	**4**	**6**
SP (survival rate, %)	82.0 ± 6.5	90.3 ± 4.5	86.3 ± 6.2
Non-SP (survival rate, %)	79.0 ± 6.0	63.0 ± 2.6	54.6 ± 1.5
HEC-1A of control group (survival rate, %)	77.3 ± 8.0	74.3 ± 7.2	60.3 ± 3.8
P^*^	0.662	0.003	0.001
P^**^	0.502	0.032	0.002
P^***^	0.807	0.097	0.308

* *Comparison between SP cells and non-SP cells*.

** *Comparison between SP cells and HEC-1A of the control group*.

*** *Comparison between non-SP cells and HEC-1A of the control group*.

#### Determination of the Sensitivity of HEC-1A-SP and HEC-1A-non-SP Cells to X-Rays Radiation Therapy

**Figure 4:**
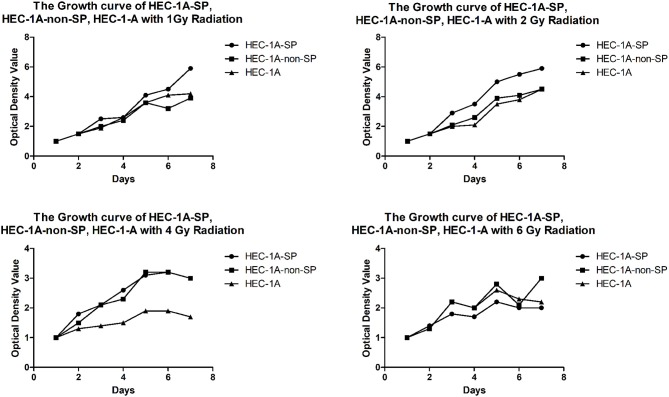
The Growth curve of HEC-1A-SP, HEC-1A-non-SP, HEC-1-A with different radiation doses (Gy).

The results of the determination of the sensitivity of HEC-1A-SP and HEC-1A-non-SP cells to X-ray radiation therapy showed that the three kinds of cells were sensitive to X-ray radiotherapy to a certain extent; as the radiation dose increased, the cell growth of each groups decreased. However, the SP cells could better tolerate the low-dose X-ray irradiation of 1 and 2 Gy than the non-SP cells and other cells, but there were no differences in the high-dose X-ray irradiation of 4 and 6 Gy, which suggested that the SP cells had a certain level of radiation resistance, as shown in Figure 4 (A formula, namely, Surviving fraction = group with irradiation/group without irradiation, was used to exclude the non-uniformity of cells during decking. All data were standardized; the light absorbance value of the first day was used as the baseline).

### Determination of the Sensitivity of Ishikawa–SP Cells to MPA

#### Growth Inhibitory and Apoptosis Effect in the SP and Non-SP Cells of the Ishikawa

##### Cell Line in response to MPA

The results of the growth inhibition of the SP and non-SP cells of Ishikawa in response to MPA as detected by MTS showed that with the increase in MPA concentration and time, the growth inhibition rates of the three kinds of cells increased (*P* < 0.05). MPA had the weakest inhibitory effect on Ishikawa-SP cells, suggesting that the SP cells may have progesterone resistance compared with the control group of Ishikawa and Ishikawa-non-SP cells, and the inhibition rate of the Ishikawa cell line treated with 10 μmol/L concentration was 50% at 72 h.

After 72 h of MPA treatment, the apoptosis rates were measured for the Ishikawa-SP, Ishikawa-non-SP and the control groups as 4.01 ± 0.43, 9.3 ± 0.67, and 4.64 ± 0.18, respectively (*P* < 0.05). The results showed significant differences between each group (see Table 2).

**Table 2:** The inhibitory rate of Ishikawa-SP, Ishikawa-non-SP, and Ishikawa cells treated with different concentrations of MPA for 72 h ($\bar{x}$ ± s).

**Items**	**MPA (μmol/L)**			
	**5**	**10**	**15**	**20**
SP (Inhibitory rate, %)	12.6 ± 3.0	13.5 ± 4.5	20.8 ±6.2	29.1 ± 5.9
Non-SP (Inhibitory rate, %)	17.4 ± 4.9	57.6 ± 2.2	68.4 ± 6	78.3 ± 1.9
Ishikawa (Inhibitory rate, %)	18.7 ± 4.0	50.8 ± 2.4	64.3 ± 3.8	74.4 ± 2.0

*Compared with the same concentration treatment of the cell groups. P < 0.05*.

#### Determination of Caspase-3 Expression in the Ishikawa-SP, Ishikawa-non-SP, and Ishikawa Cell Lines in Response to MPA Treatment

The immunocytochemistry staining results showed that there was Caspase-3 expression in the cytoplasm of the Ishikawa-SP, Ishikawa-non-SP and Ishikawa cell lines, but caspase-3 expression in the SP cells was significantly weaker than that in the non-SP cells and Ishikawa cells.

#### Determination of the Autophagy Activity in the Ishikawa-SP, Ishikawa-non-SP, and Ishikawa Cell Lines in Response to MPA Treatment

**Figure 5:**
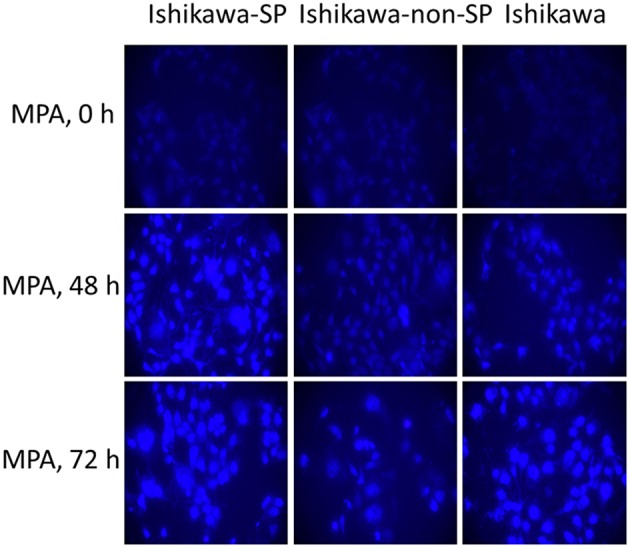
Autophagy activity detected by MDC staining.

After 0, 48, and 72 h of MPA treatment, autophagy activity was detected by MDC staining. The results showed that autophagy activity increased in the Ishikawa-SP, Ishikawa-non-SP and Ishikawa cell lines. Of the Ishikawa-SP, Ishikawa-non-SP and Ishikawa cell lines, Ishikawa-SP cells showed the strongest autophagy activity, while Ishikawa-non-SP showed the weakest autophagy activity, as shown in Figure 5.

## Discussion

Recent research has shown that tumor stem cells constitute a small group of stem cells with the capability of self-renewal and therapeutic resistance. Tumor stem cells were found in tumors of the haematopoietic system (11) and in solid tumors [e.g., breast cancer (12), brain tumors (9, 13), lung cancer (14), esophageal cancer, colorectal cancer (15), liver cancer (16), etc.]. Currently, studies on tumor stem cells have changed from the separation, purification, and cultivation of these cells to studies of their biological characteristics, including genomics and proteomics expression profiling, and the purpose was to provide new strategies for clinical treatment. At present, there are few reports and studies on endometrial stem cells. In this paper, Hoechst33342 staining was used (9) to select and separate tumor stem cells in endometrial cancer, and the chemoresistance, progestin resistance, and radioresistance of these cells were studied.

### Studies About the Separation and Basic Characteristics of Endometrial Cancer Stem Cells

Kato first isolated SP cells from the normal endometrium (17), later isolated 0.1–0.5% of SP cells from the tumor tissues of patients with endometrial cancer and the endometrial cancer HEC-1 cell line, and inoculated 1 × 10^4^ HEC-1-SP cells into nude mice, which caused the formation of tumors (18), indicating that SP cells in endometrial cancer had self-renewing and invasive abilities. This study detected the content of SP cells in endometrial cancer cell lines with different expression statuses of estrogen and progesterone receptor, and the results showed that there were different numbers of SP cells among the HEC-1A (0.3–3.0%), Ishikawa (0.2–6.5%), and RL95-2 (1.8–4.3%) cell lines and that there were no differences in the proportions of SP cells among the three kinds of cell lines, which was consistent with the results in the literature. The different contents of SP cells may be related to the degree of tumor differentiation, Hoechst33342 staining concentration, staining time, concentration of verapamil, and other factors, besides the type of tumor cell.

In this study, under a microscope, the volume of SP cells was slightly smaller than the volume of non-SP cells in the HEC-1A cell line. In conventional culture, SP cells were more likely to attach than non-SP cells and more like to grow into clones; however, few non-SP cells were attached, and a larger number of these cells were in the suspended state.

Kato et al. (18) separated SP cells in the endometrial cancer HEC-1 cell line and discovered that the non-SP cells grew faster than the SP cells within 10 days after separation; however, over the next 50 days, the growth of the non-SP cells was apparently stagnated, and there was apoptosis. In the study of thyroid cancer by Mitsutake et al. (19), a similar phenomenon was observed. This phenomenon might have multiple causes, including the asymmetric division of SP cells, the one-way or two-way acceleration of the growth of each cell type between SP and non-SP cells, or the influence of separation on cell viability. In our study, the growth curve was determined based on the MTS method, and it showed that non-SP cells grew fastest within 7 days after separation and that the doubling time was shortest; however, there was no significant difference between the growth rates of the three kinds of cells. However, after 14 days of culturing, the rate of clone formation of the SP cells was significantly higher than that of the non-SP cells, suggesting that the SP had a stronger clone forming ability and could better self-renew.

In accordance with the theory of tumor stem cells, the occurrence and development of the tumor is dependent on its tumor stem cells. SP cells have a stronger ability of self-renewal, which reflects the biological characteristics of the tumor stem cells to a certain extent, but animal experiments are still needed to verify the tumourigenicity. In this study, HEC-1A SP cells were inoculated into immunodeficient mice at 1 × 10^5^ cells per mouse; the rate of tumor formation in the SP group was 87.5%, while that of the non-SP group was only 12.5%, suggesting that the SP cells had much stronger tumourigenicity.

### Research on the Chemoresistance and Characteristics of SP Cells in Endometrial Cancer

In recent years, studies have shown that tumor stem cells have obvious resistance to conventional cytotoxic drugs and radiotherapy, which can induce DNA damage. Because tumor stem cells may be in the tumor, conventional chemotherapy, and radiotherapy may fail to kill all tumor stem cells. Therefore, these cells will become the sources of tumor recurrence and distant metastasis, and the treatment of tumor stem cells is expected to become a new target for cancer treatment.

In research of oral cancer SCC25 cell lines, it was found that SP cells showed more resistance to chemotherapy than 5-FU (20). According National Comprehensive Cancer Network, Platinum-based combined regimens as platinum-paclitaxel (TC) is usually used chemotherapy regimen for advanced and recurrent endometrial cancer. Paclitaxel (Taxol) is a first-line chemotherapeutic drug for gynecological malignancies and exerts anti-tumor effects through various mechanisms, such as blocking the mitosis of tumor cells and inducing apoptosis. There were many studies of Cisplatin-resistant in endometrial cancer. While, there were few studies have focused on the mechanism of paclitaxel-resistance in endometrial cancer. Therefore, we want to explore whether there is paclitaxel- resistance endometrial cancer cells and its mechanism. In this study, the preliminary results showed that when the SP cells and non-SP cells of the HEC-1A cell lines were compared, the SP cells had significant resistance to paclitaxel, and further detection by immunocytochemical staining showed that the drug resistance may be related to the expression of BCRP. ABCB1 and ABCG2 are the most basic multidrug resistance protein in different tumor tissues, ABCB1 encodes p-gp, and ABCG2 encodes BCRP. These transporters actively pump drugs out of cells by using the energy generated by the breakdown of ATP, thus protecting themselves from cytotoxic drugs and making them insensitive to chemotherapy. Some studies suggested that the high expression of ABCG2/BCRP in the cell membrane was the necessary condition of SP cells to discharge Hoechest33342 dye and maintain the dry characteristics of SP cells (21). Additional studies found that the SP cells with high expression of ABCG2/BCRP isolated from a variety of cell lines also had stronger drug resistance (21, 22), suggesting that drug resistance may be related to the expression of BCRP and that the relevant mechanism needs to be further studied, which is consistent with previous reports (23).

### Research on the Radioresistance Characteristics of Endometrial Cancer SP Cells

In this study, the radiosensitivity of HEC-1A cells, SP cells and non-SP cells and the preliminary results showed that the three kinds of cells were sensitive to radiotherapy up to a certain dosage. The survival rate was negatively correlated with the irradiation dosage in the mid- to low-dose radiation group, while the survival rate of the SP cells was higher, which was similar to the findings of the literature (23–26). Especially in the 2-Gy irradiation dose group, the survival rate of the SP cells was significantly higher than that of the non-SP cells and unsorted HEC-1A cells, suggesting that SP cells may be an important factor that causes radioresistance. However, in the high-dose irradiation groups, all cell growth was significantly inhibited among the three groups.

The specific mechanism underlying the radiation-resistance of stem cells is not clear. Kastan et al. (20) reported that there were many mechanisms that delayed or blocked the cell cycle for facilitating the repair of DNA when DNA was damaged. Such cycle blocking was a kind of favorable protective mechanism in normal cells, but cycle blocking may enhance the radiation-resistance of tumor cells. Multiple studies found that the expression of BCRP on the cell surface was highly related to the PI3K/Akt signaling pathway. The studies by Tappei Takada et al. (27) showed that the cell location of ABCG2/BCRP could be changed by the activity of Akt, consequently affecting the pumping capacity of ABCG2/BCRP. Generally, it is understood that the PI3K/Akt signaling pathway can be affected by the radiation-resistance of the tumor via the anti-apoptosis mechanisms and the activation of DNA repair (28). In non-small cell lung cancer, the up-regulation of the expression levels of PI3K/Akt signaling pathway mediators was related to radiation sensitivity, and cell apoptosis and cell cycle G2/M-phase blocking were induced after an Akt phosphorylation inhibitor was used (29).

In this study, the expression of BCRP in SP cells was significantly higher than that in non-SP cells, suggesting that radiation-resistance may be associated with the expression of BCRP in SP cells; however, the radiotherapy resistance mechanism of endometrial cancer stem cells (e.g., the relationship between radiation resistance and the cell cycle/signaling pathway) needs further study.

### Biological Characteristics and Mechanisms of Progesterone Resistance in Endometrial Cancer Stem Cells

Most of endometrial cancer and breast cancer are hormone-dependent tumors. Studies have shown that the expression of breast cancer resistance protein, cytokeratin 8 and pheochromocytoma A may be related to multipotential differentiation stem cells, leading to drug resistance in breast cancer cell lines (30, 31). Whether endometrial cancer shows endocrine therapy resistance that is similar to breast cancer and whether the endocrine resistance has a separate mechanism remain unknown. Therefore, the study of endometrial cancer stem cell progesterone resistance may provide the basis for endocrine cancer endocrine therapy.

Zhao and Liu et al. (32, 33) carried out drug resistance studies of endometrial cancer cell lines by gradually increasing the MPA concentration *in vitro*, and as a result, progesterone-resistant cell lines of endometrial cancer were successfully established. Combined with reports in the literature, studies have shown that the mechanism of endometrial cancer resistance to progesterone may be related to an imbalance in the expression of progesterone receptor subtypes and to the abnormal expression or sustained activation of epidermal growth factor receptor (EGFR) and the activation its downstream signaling pathways. Resistance may also be associated with PR-A mRNA downregulation, PR-B/PR-A mRNA upregulation, and PR protein expression increases, which cause progesterone resistance in the endometrium (34). Cancer stem cells have a more powerful ability to repair DNA than the differentiated cells of the tumor, which makes tumor stem cells more adaptable to various changes in the environment, to ensure prompt repair of tumor tissue after injury and to favor the occurrence of drug resistance.

At present, there are few reports on endometrial cancer in terms of progesterone resistance or drug resistance. In this study, Hoechst33342 staining was used to isolate tumor stem cells, namely, SP cells, in endometrial carcinoma, and the cells were treated with different concentrations of MPA. The results showed that MPA had the lowest growth inhibitory effect in Ishikawa-SP cells. Apoptosis assays by flow cytometry showed that MPA treatment resulted in the lowest apoptotic rate of Ishikawa-SP cells compared with Ishikawa and Ishikawa-non-SP cells. The results of immunocytochemistry further showed that the expression of apoptotic protein casepase-3 in Ishikawa-SP was the weakest after MPA treatment. It is suggested that endometrial cancer stem cells with progesterone may be related to anti-apoptosis after MPA. The preliminary results suggest that the mechanism of progesterone resistance in endometrial cancer stem cells may be related to apoptosis, but the mechanism of action still needs further investigation.

## Conclusion

In conclusion, this study showed that there were few SP cells in differentiated endometrial cancer cell lines, and the study of the biological characteristics of SP cells in the HEC-1A cell line showed that there was the capacity for clone formation, chemoresistance, progesterone resistance, and radioresistance *in vitro*. These observations still need to be further explored for providing a basis for the study of drug resistance and radioresistance to decreased disease recurrence.

## Data Availability Statement

The datasets used and analyzed during the current study are available from the corresponding author on reasonable request.

## Ethics Statement

This study was approved by the Ethics Committees of Peking University Peoples Hospital (No.2018PHC054).

## Author Contributions

XL, JW, and LW conceived the experiments. BL, QX, WY, NL, TY, and LZ conducted the experiments. QX, WY, NL, TY, and LZ analyzed and interpreted the data. BL and QX wrote and prepared the manuscript and the figures. All authors read and approved the final manuscript.

### Conflict of Interest

The authors declare that the research was conducted in the absence of any commercial or financial relationships that could be construed as a potential conflict of interest.
